# Bone marrow infiltrated natural killer cells predicted the anti-leukemia activity of MCL1 or BCL2 inhibitors in acute myeloid leukemia

**DOI:** 10.1186/s12943-020-01302-6

**Published:** 2021-01-05

**Authors:** Yu-Jun Dai, Si-Yuan He, Fang Hu, Xue-Ping Li, Jian-Ming Zhang, Si-Liang Chen, Wei-Na Zhang, Hai-Min Sun, Da-Wei Wang

**Affiliations:** 1 Department of Hematologic Oncology, Sun Yat-sen University Cancer Center, Guangzhou, 500020 China; 2 State Key Laboratory of Oncology in South China, Collaborative Innovation Center for Cancer Medicine, 651 Dongfeng East Road, Guangzhou, 500020 China; 3 The University of Texas MD Anderson Cancer Center UTHealth Graduate School of Biomedical Sciences, Houston, TX 77030 USA; 4 National Research Center for Translational Medicine, Ruijin Hospital affiliated to Shanghai Jiao Tong University School of Medicine, Shanghai, 200025 China; 5 Department of Hematology, Peking University Shenzhen Hospital, Shenzhen, 518036 China; 6 Department of Hematology, Guangzhou Women and Children’s Medical Center, Guangzhou Medical University, Guangzhou, 510623 China; 7 State Key Laboratory of Medical Genomics, Shanghai Institute of Hematology, Ruijin Hospital affiliated to Shanghai Jiao Tong University School of Medicine, Shanghai, 200025 China; 8 Department of Hematology, Rui-Jin Hospital North, Shanghai Jiao Tong University School of Medicine, Shanghai, 201800 China

**Keywords:** NK cells, MCL1 inhibitor, BCL2 inhibitor, AML, Immunotherapy

## Abstract

Acute myeloid leukemia (AML) is still incurable due to its heterogeneity and complexity of tumor microenvironment. It is imperative therefore to understand the molecular pathogenesis of AML and identify leukemia-associated biomarkers to formulate effective treatment strategies. Here, we systematically analyzed the clinical characters and natural killer (NK) cells portion in seventy newly-diagnosis (ND) AML patients. We found that the proportion of NK cells in the bone marrow of ND-AML patients could predict the prognosis of patients by analyzing the types and expression abundance of NK related ligands in tumor cells. Furthermore, MCL1 inhibitor but not BCL2 inhibitor combined with NK cell-based immunotherapy could effectively improve the therapeutic efficiency via inhibiting proliferation and inducing apoptosis of AML primary cells as well as cell lines in vitro. There results provide valuable insights that could help for exploring new therapeutic strategies for leukemia treatment.

**Supplementary Information:**

The online version contains supplementary material available at 10.1186/s12943-020-01302-6.

## Main text

Natural killer (NK) cells are a type of cytotoxic immune cells that can recognize and kill cancer cells rapidly and efficiently. In recent years, more and more studies have revealed the biological characteristics of NK cells and their ability to recognize cancer cells directly [1]. Although immunotherapy has made a great breakthrough in stimulating the immune system against hematologic malignancies, there are few studies on NK cell-based immunotherapy [2].

Killer cell immunoglobulin-like receptors (KIR) are a type of receptor mainly expressed on the surface of human NK cells and partially activated T cells [3]. Functionally, KIR genes could be divided into inhibitory and activated types, which can specifically recognize and bind HLA class I molecules on the surface of target cells. They regulate the killing function of active cells in an effective switch system and play an important role in anti-infection and anti-tumor [4].

In this study, we elucidated the clinical relevance of KIRs and NK cells in bone marrow (BM) of acute myeloid leukemia (AML) patients. Our data indicated that NK cell ratio can predict the prognosis of patients, and can synergistically kill leukemia cells with MCL1 inhibitors to improve treatment efficiency.

## Results and discussions

### Expression level and prognosis of KIRs in AML

**Fig. 1:**
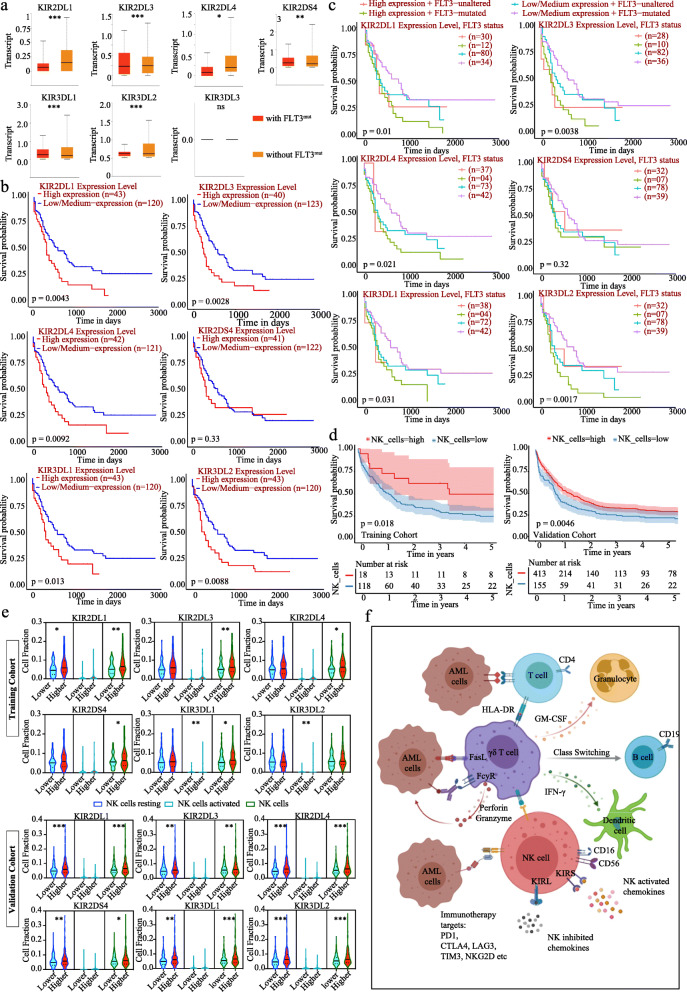
Overall survival (OS) of AML patients based on their BM infiltrated NK cells and its receptors KIRs. **a** KIRs expression levels in patients with or without FLT3 mutations. **b** Kaplan-Meier plots of OS for patients according to the expression level of KIRs, respectively. **c** The prognostic values of the KIRs factors combined with FLT3 mutation status. **d** OS of patients according to the BM infiltrated NK cells in training (4 missing cases) and validation cohorts (10 missing cases). **e** The expression of KIRs were in groups with high or low NK cells, activated and resting NK cells, respectively. Resting NK cells is colored in blue, activated NK cells in Turquoise, and bulk NK cells in green. Mean ± SEM values are shown. *P < 0.05, **P < 0.01, ***P < 0.001. **f** A proposed cellular model to describe the function of immune cells in patients

Here we reported a study on the transcriptional levels of KIRs in cancer and normal samples by analyzing the data from Oncomine and ENCORI (The Encyclopedia of RNA Interactomes) (Additional file 1 and Table S1). These results indicated that KIRs played an important role in solid tumors such as kidney renal clear cell carcinoma and lung cancer. In AML patients, the expression levels of KIRs were much higher than that in normal samples (Additional file 2). Notably, the expression of KIR2DL group (KIR2DL1, KIR2DL3 and KIR2DL4) was significantly downregulated in patients with FLT3 mutations, whereas KIR2DS and KIR3DL group (except KIR3DL3) were upregulated (Fig. 1a). Interestingly, RAS activation status was not related to the expression of KIRDS in AML (Additional file 3). Next, we further explored the critical efficiency of KIRs for predicting the survival of patients with AML. The Kaplan-Meier curve and log rank test analyses revealed that the increased KIR2DL1 (*p* = 0.0043), KIR2DL3 (*p* = 0.0028), KIR2DL4 (*p* = 0.0092), KIR3DL1 (*p* = 0.013) and KIR3DL2 (*p* = 0.0088) mRNA levels were significantly related to poor prognosis for overall survival (OS) of AML patients (Fig. 1b). Whereas, the KIR2DS4 mRNA level had no tendency to indicate prognosis (*P* = 0.33). Furthermore, when FLT3 mutation status was combined, the prognostic values of the KIRs factors were consistent with the above results (Fig. 1c).

### NK cells in BM indicated poor prognosis in AML

The immune dysfunction recently has been considered as a risk factor in AML and predicted poor prognosis [5]. Next, we utilized Cibersort to deconvolute the gene-expression data of 713 newly diagnosed AML (ND-AML) patients (140 patients as Training cohort and 573 patients as Validation cohort) and generated a gene matrix with a signature of more than 10 immune cell subtypes (Table S2 and S3). Patients were divided into “low” and “high” subgroups (according to the cutoff value of conversion score of total NK cells, activated NK cells and resting NK cells, respectively). The prognostic analysis indicated that the low NK cells or low resting NK cells predict poor prognosis, while the low activated NK cells indicated a favorable prognosis in AML patients (Fig. 1d and Additional file 4). Notably, the differential expression of KIRs were only in total NK cells but not in activated or resting NK cells (Fig. 1e). Thus, we proposed a model to describe the cellular and molecular basis for the potential prognostic value of monitoring the proportion of immune cells in patients (Fig. 1f).

**Fig. 2:**
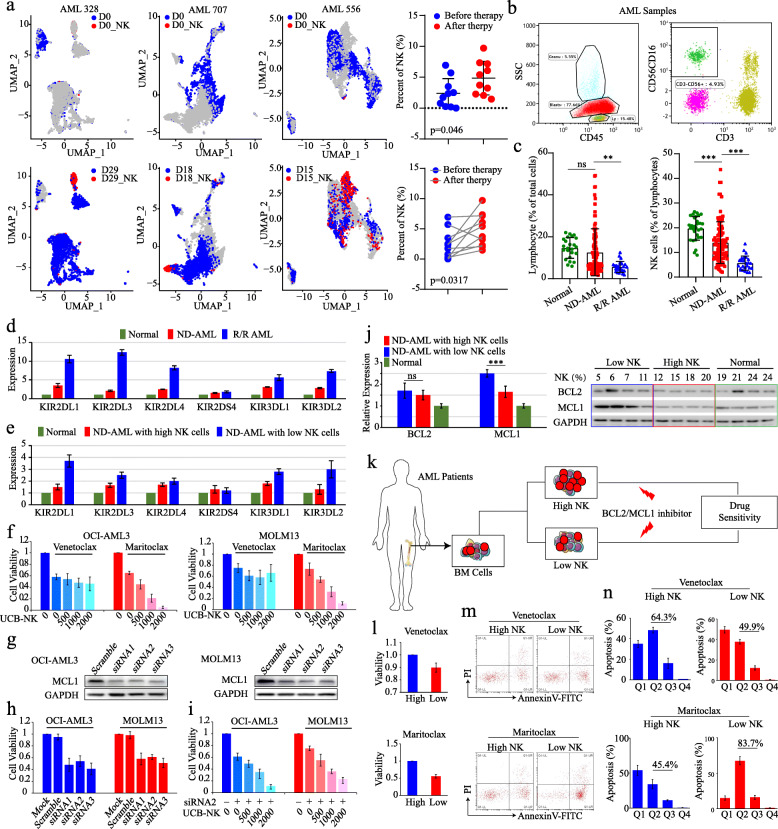
NK cells enhanced the anti-leukemia activity synergized with MCL1 inhibitor. **a** UMAP analysis of NK cells in AML samples at diagnosis and after chemotherapy by using single-cell RNA sequence data. The percentage of NK cells among them were further compared. **b** The flow cytometry analysis of BM infiltrated NK cells by CD45^+^CD3^−^CD56^+^CD16^+^ gating. **c** Proportions of lymphocytes and NK cells subsets in BM of patients with lymphoma, ND-AML and R/R AML. **d** The expression levels of KIRs in BM of patients with lymphoma, ND-AML and R/R AML. **e** Comparison of KIRs expression levels between high NK group and low NK group in ND-AML patients. **f** The cell viability test of OCI-AML3 and MOLM13 treated with venetoclax or maritoclax by co-cultured with UCB-NK cells. **g** Protein validation of the knockdown efficiency of MCL1 siRNAs in OCI-AML3 and MOLM13 cells. **h-i** The cell viability of OCI-AML3 and MOLM13 treated with MCL1 siRNA or scramble siRNA (**h**) and co-cultured with UCB-NK cells (**i**). **j** RNA and protein expression levels of BCL2 and MCL1 in normal controls and ND-AML samples with low or high NK cells. **k** Scheme of cell viability analysis by using primary samples of AML patients. **l** Cell viability of BM cells with high or low NK cells with venetoclax or maritoclax treatment. **m-n** Apoptosis induced by inhibitors among AML patient cells with high or low NK cells. Mean ± SEM values are shown. *P < 0.05, **P < 0.01, ***P < 0.001

Further, the single-cell RNA sequence data of AML patients at diagnosis and matched samples after chemotherapy were used for immune cell subtype analysis [6]. UMAP (Uniform Manifold Approximation and Projection) analysis indicated the proportion of NK cells in total BM cells of AML samples at diagnosis was much lower than those in matched samples after chemotherapy (Fig. 2a). We further validated the NK cells proportion (CD45^+^CD3^−^CD56^+^CD16^+^) of lymphocytes in BM cells from patients with hematological malignancies (30 lymphoma cases without BM infiltration as control; 95 ND-AML cases and 25 refractory/relapse (R/R) AML cases) (Fig. 2b). The proportion of lymphocytes, especially NK cells in BM, was significantly decreased in ND-AML samples compared with normal samples, and the ratio reached the lowest in R/R AML cases (Fig. 2c). These data were consistent with theory that NK cells might be one of important mediators of anti-leukemia immunity and indicated that NK cells of BM might play an anti-leukemia effect in leukemogenesis [7, 8]. In addition, KIRs expressions were much higher in R/R AML samples among these three groups (Fig. 2d and Table S4). When we divided ND-AML samples into high NK cells group and low NK cells group, according to the proportion of NK cells in BM. We found there was no significant differences in the clinical characteristics of the two groups (Table 1). However, KIRs were mainly expressed in samples with low NK cell group (Fig. 2e and Table S4). The expression data further validated survival data of NK cells and KIRs expression patterns which showed above.

**Table 1:** Basic characteristics of ND-AML patients between NK% high and NK% low in BM

Variable	ND-AML NK%^**high**^ (***n***=34)	ND-AML NK%^**low**^ (***n***=35)	***P*** value
**Age, years**			ns
Median		47	
Range	15–78	19–74	
**Gender, no. (%)**			ns
Male	20 (58.8%)	16 (45.7%)	
Female	14 (41.2%)	19 (54.3%)	
**Hepatic or renal function, no.**^a^			ns
Normal	32	32	
Abnormal	2	3	
**WBC, 10**^**9**^**/L**			ns
Median	10.665	13.79	
Range	1–249.98	1.4–371.9	
**Platelet, 10**^**9**^**/L**			ns
Median	34.5	28	
Range	1.87–401	3–167	56
**HB, g/L**			ns
Median	81	74	
Range	20–116	52–149	
**PB NK, %**			< 0.001
Median	14.1	5.4	
Range	4.2–42.9	2.3–21.7	
**BM blasts, %**			ns
Median	53.7	53.1	
Range	6.6–94.1	16.3–95.1	
**BM Lymph, %**			ns
Median	11.3	6.2	
Range	2.2–49	1.7–49.2	
**BM NK, %**			< 0.001
Median	19.65	7.6	
Range	12.5–43.6	2–11.9	
**BM T, %**			< 0.001
Median	66.5	77.7	
Range	45.6–80.4	46–86.2	
**Cytogenetics, no. (%)**			
t(15;17)/PML-RARA	0	0	
t(8;21)/AML1-ETO	1	4	
inv.(16;16)/CBFb-MYH11	2	1	
CN-AML^b^	23	22	
Unfavorable^c^	8	8	
**Mutations, no. (%)**			
FLT3 mutations	15	5	
RAS mutations	6	6	

*ALT* Alanine aminotransferase, *AST* Aspartate aminotransferase, *WBC* White blood cell, *RBC* Red blood cell, *HB* Hemoglobin, *BM* Bone marrow, *ns* Not significance

*P* values were calculated by means of nonparametric Wilcoxon rank-sum test for continuous variables and x-square test for categorical variables

^a^Hepatic abnormality as defined by ALT 2.53 normal value or AST 2.53 normal value, while renal abnormality as defined by creatinine 2.53 normal value

^b^CN-AML: cases having no cytogenetically identifiable abnormalities

^c^Unfavorable: inv. (3)/t (3;3), t (9;22), 11q23 abnormalities, 25, 27, del (5q),del (7p), and complex karyotype

### NK cells could synergy with MCL1 inhibitor to kill leukemia cells

Previous studies showed that BCL-2 and MCL-1 proteins could control the survival of NK cells in vivo [9]. Thus, we explored the relationship between the expression of these two molecules and NK cells in BM. We found MCL1 expression level has a significant negative correlation with NK cells, while BCL2 expression level has no correlation with NK cells both in training and validation cohorts (Additional file 5). As we known, BCL2 and MCL1 inhibitors are currently considered as new therapeutics for leukemia, and showed promising response in patients [10]. Therefore, we quest whether NK cells could synergize with these new inhibitors to achieve better therapeutic effects. To test our hypothesis, we treated AML cell lines OCI-AML3 and MOLM13 with BCL2 inhibitor (Venetoclax) or MCL1 inhibitor (Maritoclax), then co-cultured with gradient numbers of NK cells derived from umbilical cord blood (UCB-NK). The cell viability suggested that UCB-NK could cooperate with maritoclax but not venetoclax to kill leukemia cells (Fig. 2f). Moreover, we applied the MCL1 siRNA to knockdown the expression of MCL1 in OCI-AML3 and MOLM13 cells and then cocultured with UCB-NK (Fig. 2g-i). The cell viability was significantly decreased in both OCI-AML3 and MOLM13 cells treated with MCL1 siRNA compared with those treated with scramble siRNA (Fig. 2h). In addition, the cell viability of OCI-AML3 and MOLM13 was significantly impaired after treatment with MCL1 siRNA and UCB-NK cells (Fig. 2i). This result was consistent with the data on the pharmacological effects of maritoclax (Fig. 2f). Next, we sought to validate effects by using mononuclear cells isolated from the BM of ND-AML patients. Samples were divided into two groups according to the NK proportion in BM (Fig. 2k and Table S4), and the RNA/protein expression levels of BCL2 and MCL1 were examined. MCL1 was much more expressed in the low NK cell group of ND-AML samples both at transcriptional and translational level (Fig. 2j). Consistent with the results from cell line studies, patient samples with low NK treated with maritoclax were less viable than samples with high NK (Fig. 2l). In addition, maritoclax induced cell apoptosis was significant enhanced in samples with low NK proportion (Fig. 2m and n). While there was no significant difference in cell viability and apoptosis between samples with high NK and low NK with venetoclax treatment (Fig. 2k-n).

## Conclusions

The importance of NK ratio in BM of AML patients has generated tremendous interest in understanding its role in disease management. Immune dysfunction could predict therapeutic reactivity and unfavorable prognosis [5]. By analyzing the types and expression abundance of ligands related to NK function expressed in tumor cells, we first proposed that the proportion of NK cells in the BM of AML patients could predict the prognosis of patients. The lower the proportion of NK cells, the worse of the prognosis. Therefore, increasing the number of NK cells through small molecule compounds or cytokines, such as interleukin 15 or interleukin 2 etc., may be a new method of anti-leukemia. Next, we plan to perform experiments to verify the effect of NK on prognosis in different conditions, and conduct prospective controlled cohort clinical trials to verify this hypothesis in the future. Second, our data indicated that NK cell-based immunotherapy combined with MCL1 inhibitor but not BCL2 inhibitor could effectively improve the therapeutic efficiency of AML. These findings might provide new insights and theoretical basis for exploring new targets for leukemia treatment.

## Supplementary Information

**Additional file 1.**

**Additional file 2.**

**Additional file 3.**

**Additional file 4.**

**Additional file 5.**

**Additional file 6.**

**Additional file 7.**

**Additional file 8.**

**Additional file 9.**

**Additional file 10.**

## Data Availability

All the data obtained and/or analyzed in the current study were available from the corresponding authors on reasonable request.

## Abbreviations

NK: Natural killer
KIR: Killer cell immunoglobulin-like receptors
HLA: Human leukocyte antigen;
ENCORI: The Encyclopedia of RNA Interactomes
AML: Acute Myeloid Leukemia
ND-AML: Newly diagnosed AML
UMAP: Uniform Manifold Approximation and Projection
R/R AML: Refractory/relapse AML
UCB-NK: NK cells derived from umbilical cord blood
