# Supplementary material for: Bone marrow infiltrated natural killer cells predicted the anti-leukemia activity of MCL1 or BCL2 inhibitors in acute myeloid leukemia
Source: Mol Cancer. 2021 Jan 5;20:8. doi: 10.1186/s12943-020-01302-6 (PMC7784307; doi:10.1186/s12943-020-01302-6)
Supplement: Supplementary file 1 — Additional file 1. [file 12943_2020_1302_MOESM1_ESM.pdf]

Analysis Type by Cancer	Cancer vs. Normal		Cancer vs. Normal		Cancer vs. Normal		Cancer vs. Normal		Cancer vs. Normal		Cancer vs. Normal		Cancer vs. Normal	
	KIR2DL1		KIR2DL3		KIR2DL4		KIR2DS4		KIR3DL1		KIR3DL2		KIR3DL3	
Bladder Cancer														
Brain and CNS Cancer														
Breast Cancer			1		1									
Cervical Cancer														
Colorectal Cancer		1				4		1				1		
Esophageal Cancer				2										
Gastric Cancer		1				1								
Head and Neck Cancer														
Kidney Cancer	2									2				
Leukemia					1				1		4	1		
Liver Cancer														
Lung Cancer				1								1		
Lymphoma		1		2						1	1	1		
Melanoma											1			
Myeloma														
Other Cancer										2				
Ovarian Cancer														
Pancreatic Cancer						1								
Prostate Cancer														
Sarcoma	1				1	1			1	3		3		
Significant Unique Analyses	3	3	1	5	3	7		1	2	7	5	7		
Total Unique Analyses	321		382		378		142		382		367		139	

1 5 10 10 5 1

%

Cell color is determined by the best gene rank percentile for analyses within the cell.

NOTE: An analysis may be counted in more than one cancer type.

Analysis Type by Cancer	Cancer vs. Normal		Cancer vs. Normal		Cancer vs. Normal		Cancer vs. Normal		Cancer vs. Normal		Cancer vs. Normal		Cancer vs. Normal	
	KIR2DL1		KIR2DL3		KIR2DL4		KIR2DS4		KIR3DL1		KIR3DL2		KIR3DL3	
Bladder Cancer														
Brain and CNS Cancer														
Breast Cancer			1		1									
Cervical Cancer														
Colorectal Cancer		1				4		1				1		
Esophageal Cancer				2										
Gastric Cancer		1				1								
Head and Neck Cancer														
Kidney Cancer	2									2				
Leukemia					1				1		4	1		
Liver Cancer														
Lung Cancer				1								1		
Lymphoma		1		2						1	1	1		
Melanoma											1			
Myeloma														
Other Cancer										2				
Ovarian Cancer														
Pancreatic Cancer						1								
Prostate Cancer														
Sarcoma	1				1	1			1	3		3		
Significant Unique Analyses	3	3	1	5	3	7		1	2	7	5	7		
Total Unique Analyses	321		382		378		142		382		367		139	

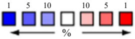

Cell color is determined by the best gene rank percentile for analyses within the cell.
NOTE: An analysis may be counted in more than one cancer type.
