# Supplementary figures and images for: Bone marrow infiltrated natural killer cells predicted the anti-leukemia activity of MCL1 or BCL2 inhibitors in acute myeloid leukemia
Source: Mol Cancer. 2021 Jan 5;20:8. doi: 10.1186/s12943-020-01302-6 (PMC7784307; doi:10.1186/s12943-020-01302-6)

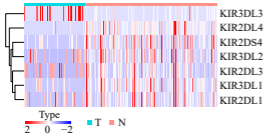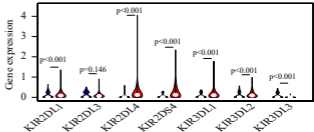

Supplement: Supplementary file 2 — Additional file 2. [file 12943_2020_1302_MOESM2_ESM.pdf]

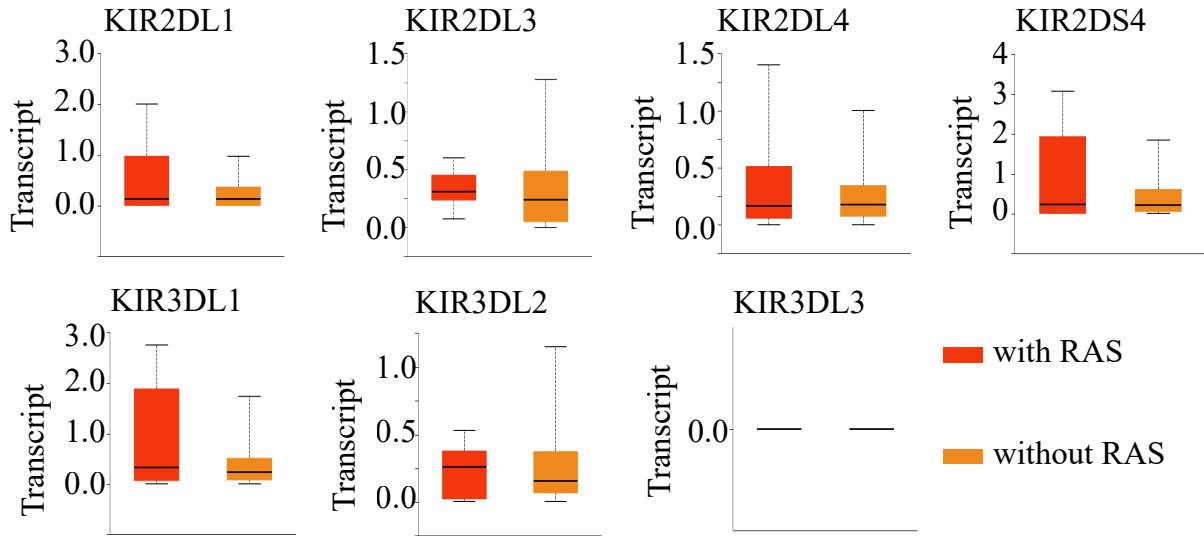

Supplement: Supplementary file 3 — Additional file 3. [file 12943_2020_1302_MOESM3_ESM.pdf]

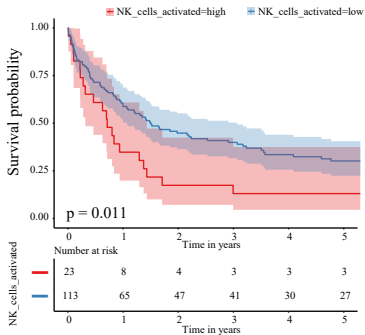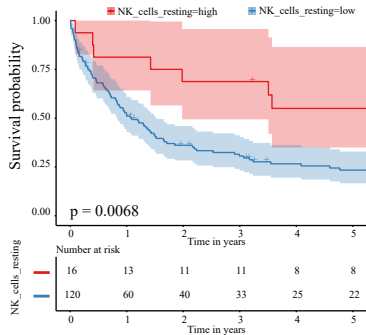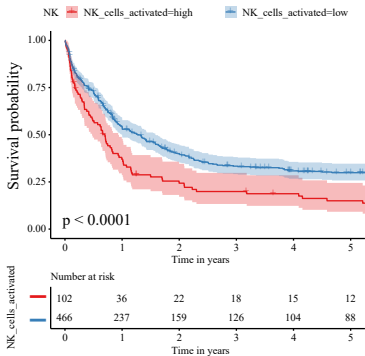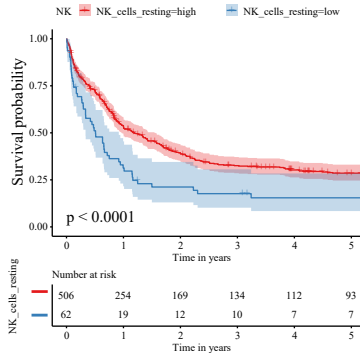

Supplement: Supplementary file 4 — Additional file 4. [file 12943_2020_1302_MOESM4_ESM.pdf]

### Training Cohort

---

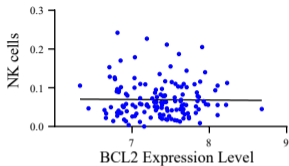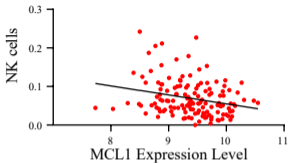

### Validation Cohort

---

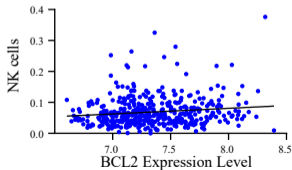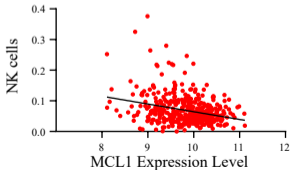

Supplement: Supplementary file 5 — Additional file 5. [file 12943_2020_1302_MOESM5_ESM.pdf]
