# Supplementary material for: Bone marrow infiltrated natural killer cells predicted the anti-leukemia activity of MCL1 or BCL2 inhibitors in acute myeloid leukemia
Source: Mol Cancer. 2021 Jan 5;20:8. doi: 10.1186/s12943-020-01302-6 (PMC7784307; doi:10.1186/s12943-020-01302-6)
Supplement: Supplementary file 10 — Additional file 10. [file 12943_2020_1302_MOESM10_ESM.docx]

**Method**

**Patients**

We enrolled 95 patients with newly diagnosed AML (ND-AML), 30 lymphoma and 25 refractory/relapse (R/R) AML samples from two center Sun Yat-sen University Cancer Center and Ruijin Hospital affiliated to Shanghai Jiao Tong University School of Medicine in this study. All enrolled participants provided written informed consent in accordance with the regulations of the Institutional Review Boards of the Sun Yat-sen University Cancer Center and Ruijin Hospital in agreement with the Declaration of Helsinki. The human cancer tissues used in this study were with the code number GZR2020-152 approved by Ethnics Committee of Sun Yat-sen University Cancer Center.

**Immune Cytotypes Conversion**

Cibersort was used to deconvolute GEO datasets (GSE37642) and TCGA database from 713 ND-AML patients and generated a gene matrix with a signature of more than 10 immune cell subtypes according to instructions (https://cibersort.stanford.edu/manual.php). Then, the cases were divided into “low” and “high” subgroups (according to conversion score of NK cells, activated NK cells and resting NK cells, respectively). The prognostic values of NK cells, activated NK cells and resting NK cells according to the cutoff value of conversion scores were analyzed by Kaplan-Meier method in AML patients.

**Flow Cytometric Analysis**

BM cells were suspended in FACS buffer (PBS with 1% FBS) and stained with fluorochrome-conjugated antibodies: CD45-Krome orange (Beckman A96416), CD56-FITC (BD 340410), CD3-APC (BD 340440), CD138-APC (Beckman A87787), CD16-FITC (Beckman IM0814U). Flow cytometric data were collected using an LSR II flow cytometer (Becton Dickinson) and analyzed with FlowJo software (TreeStar).

**Quantitative Real-Time PCR**

The expression of the KIRs was measured by quantitative RT-PCR. GAPDH was used as the internal control and the formula 2^−ΔΔCt^ was used to analyze the data. The assay was performed according to instructions of the manufacturer (ESscience; QP002). All primers are listed in supplementary Table 4.

**Single-cell RNA Sequence Analysis**

The single-cell RNA sequence data of patients with AML at diagnosis and matched samples after chemotherapy from GSE116256 was used for immune cell subtype analysis. UMAP (Uniform Manifold Approximation and Projection) analysis indicated the visible proportion of NK cells in total BM cells of AML samples at diagnosis and matched samples after chemotherapy.

**NK Cells Derived from Umbilical Cord Blood (UCB-NK)**

The UCB-NK samples were obtained from the China Cord Blood Bank of Shandong province. The incremental UCB-NK cells were cultured in RPMI-1640 (Gibco, NY) with 10% FBS (Biochrom AG, Berlin, Germany) and human interleukin-12 (IL-12, PeproTech) and interleukin-15 (IL-15, PeproTech), and co-cultured with OCI-AML3 and MOLM13 cells in 96-well plates.

**Cell Viability and Apoptosis Assay**

BCL2 inhibitor Venetoclax (S8048) and MCL1 inhibitor Maritoclax (S7126) were obtained from Selleck Chemicals LLC (Houston, TX). OCI-AML3 and MOLM13 cells (gifted from laboratory of Da-Wei Wang) were cultured and suspended in RPMI-1640 (Gibco, NY) with 10% FBS (Biochrom AG, Berlin, Germany) in 96-well plates with 10^5^ cells/well. The cytotoxicity of Venetoclax and Maritoclax was determined by cell counting kit-8 (CCK-8, Dojindo Laboratories, Kumamoto, Japan) after incubating 48 h and measured by the absorbance at 450 nm. Next, the apoptotic cells induced by Venetoclax or Maritoclax was detected by an Annexin V FITC/PI staining kit (FA111-02, Transgen Biotech).

**siRNA Transfection Assay**

Leukemia cell lines OCI-AML3 and MOLM13 were cultured in RPMI-1640 with 10% FBS and without antibiotics for 24 hours. Then, cells were transfected with MCL1 or scramble siRNA oligomer by using Lipofectamine 3000 Transfection Reagent (Invitrogen) according to the manufacturer’s instructions. Eight hours later, we changed the fresh medium with 10% FBS and antibiotics and incubated these cells at 37 ℃ for 48 hours. The sequence of MCL1 and scramble siRNA were listed:

Negative control, sense: 5’-ACGUGACACGUUCGGAGAATT-3’, antisense: 5’-UUCUCCGAACGUGUCACGUTT-3’;

hMCL1-1#, sense: 5’-UUGAUGUCCAGUUUCCGAAGCTT-3’, antisense: 5’-GCUUCGGAAACUGGACAUCAATT-3’;

hMCL1-2#, sense: 5’-UAUGGUCUUCAAGUGUUUAGCTT-3’, antisense: 5’-GCUAAACACUUGAAGACCAUATT-3’;

hMCL1-3#, sense: 5’-UUAGAUAUGCCAAACCAGCUCTT-3’, antisense: 5’-GAGCUGGUUUGGCAUAUCUAATT-3’;

**Western Blot Analysis**

Antibodies were purchased from Cell Signaling Technology (anti-BCL2), Abcam (anti-MCL1), and Santa Cruz (anti-GAPDH).

**Expression Levels and Prognostic Values of KIRs**

The expression levels of KIRs in different type of cancers were analyzed by a cancer microarray website named Oncomine and ENCORI (The Encyclopedia of RNA Interactomes). In addition, KIRs expression levels were compared in AML and normal samples by using the RNA sequencing data of The Cancer Genome Atlas (TCGA) and the Genotype Tissue Expression projects (GTEx). The “edgeR” package was used to analyze detailed expression levels of KIRs between tumor and normal samples by R studio. Moreover, the transcription expression levels of KIRs were confirmed by using UALCAN database. The cutoff value was calculated by using “survminer” package. The prognostic values of KIRs according to corresponding expression levels were analyzed by Kaplan-Meier method in AML patients from UALCAN database. All the statistics were analyzed by Student’s t test.

**Co-expression Relationship Analysis**

The “corrplot” package was utilized to analyze the co-expression index between KIRs expression levels and NK cells by Pearson analysis via R studio. Furthermore, the relationship of BCL2 or MCL1 expression level and NK cells was analyzed by Spearman and Pearson analysis using GraphPad Prism 8 software.
